# Gastric Aspirate Isolate Demonstrates Strain-Level Concordance With Sputum Isolate in Nontuberculous Mycobacterial Pulmonary Disease

**DOI:** 10.1093/ofid/ofag175

**Published:** 2026-03-27

**Authors:** Atsushi Funauchi, Kazuki Hashimoto, Kiyoharu Fukushima, Yuki Matsumoto, Nanako Hamada, Reina Hara, Takayuki Niitsu, Takuro Nii, Takanori Matsuki, Kazuyuki Tsujino, Keisuke Miki, Atsushi Kumanogoh, Shota Nakamura, Hiroshi Kida

**Affiliations:** Department of Respiratory Medicine and Clinical Immunology, Graduate School of Medicine, The University of Osaka, Suita, Osaka, Japan; Department of Respiratory Medicine and Clinical Immunology, Graduate School of Medicine, The University of Osaka, Suita, Osaka, Japan; Department of Respiratory Medicine and Clinical Immunology, Graduate School of Medicine, The University of Osaka, Suita, Osaka, Japan; Department of Respiratory Medicine, National Hospital Organization (NHO), Osaka Toneyama Medical Center, Toyonaka, Osaka, Japan; Department of Infection Metagenomics, Research Institute for Microbial Diseases, The University of Osaka, Suita, Osaka, Japan; Department of Infection Metagenomics, Research Institute for Microbial Diseases, The University of Osaka, Suita, Osaka, Japan; Laboratory of Host Defense, World Premier International Research Center Initiative Immunology Frontier Research Center (WPI-IFReC), The University of Osaka, Osaka, Japan; Department of Respiratory Medicine, National Hospital Organization (NHO), Osaka Toneyama Medical Center, Toyonaka, Osaka, Japan; Department of Respiratory Medicine, Osaka General Medical Center, Osaka, Japan; Department of Respiratory Medicine, National Hospital Organization (NHO), Osaka Toneyama Medical Center, Toyonaka, Osaka, Japan; Department of Respiratory Medicine, National Hospital Organization (NHO), Osaka Toneyama Medical Center, Toyonaka, Osaka, Japan; Department of Respiratory Medicine, National Hospital Organization (NHO), Osaka Toneyama Medical Center, Toyonaka, Osaka, Japan; Department of Respiratory Medicine, National Hospital Organization (NHO), Osaka Toneyama Medical Center, Toyonaka, Osaka, Japan; Department of Respiratory Medicine and Clinical Immunology, Graduate School of Medicine, The University of Osaka, Suita, Osaka, Japan; Department of Infection Metagenomics, Research Institute for Microbial Diseases, The University of Osaka, Suita, Osaka, Japan; Department of Respiratory Medicine, National Hospital Organization (NHO), Osaka Toneyama Medical Center, Toyonaka, Osaka, Japan

**Keywords:** gastric aspirate, nontuberculous mycobacteria, tuberculosis, variable number tandem repeat, whole-genome sequencing

## Abstract

The nontuberculous mycobacteria (NTM) isolated from gastric aspirate have demonstrated >85% strain concordance with those from the sputum, suggesting that they originate from the lungs rather than the environment. Gastric aspirate, although not yet internationally recognized, may be a useful supplementary specimen for diagnosing NTM pulmonary disease.

## Graphical Abstract

**Graphical Abstract:**
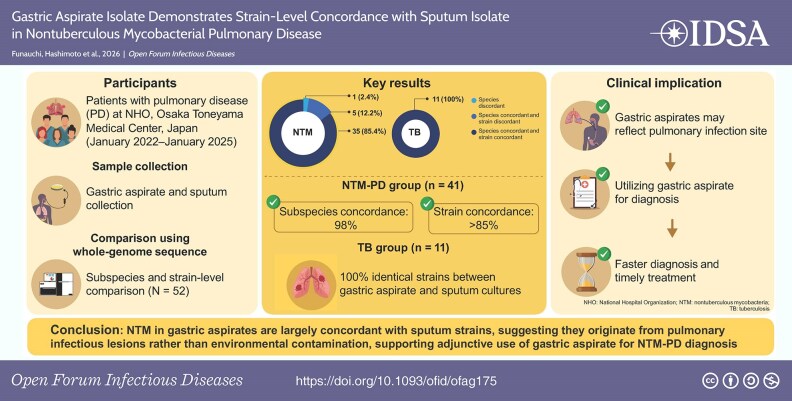
This graphical abstract is also available at Tidbit: https://tidbitapp.io/tidbits/gastric-aspirate-isolate-demonstrates-strain-level-concordance-with-sputum-isolate-in-nontuberculous-mycobacterial-pulmonary-disease-42920708-a661-4489-a4bc-9cd63bcc07a0?utm_campaign=tidbitlinkshare&utm_source=ITP

Diagnosing infectious diseases requires identifying the characteristic clinical features of the illness and confirming the presence of the causative pathogen at the site of infection. The diagnosis of nontuberculous mycobacterial pulmonary disease (NTM-PD) is based on clinical, radiologic, and microbiological criteria [1]. Importantly, the microbiological criteria for NTM-PD diagnosis mandate 2 positive sputum cultures because NTM are ubiquitous in the environment, such as soil and tap water, and contamination or transient airway colonization should be ruled out during testing. Sputum acid-fast bacilli culture has good diagnostic performance for NTM-PD [2]. However, the diagnostic yield may be limited in some patients with NTM-PD, particularly in cases of early-stage disease or in those who have difficulty expectorating sputum. Therefore, patients often need to submit many sputum samples or undergo bronchoscopy [3], resulting in a low adherence rate to the above criterion [4].

The adjunctive use of gastric aspirate culture is helpful in overcoming these issues. Gastric aspirate obtained before breakfast contains respiratory secretions swallowed during sleep [5]. In Japan, gastric aspirate culture is widely used in routine clinical practice for the diagnosis of tuberculosis in both adults and children. The US and European guidelines recommend gastric aspirate culture in children in whom sputum collection is difficult [6, 7]. Recently, the diagnostic utility of gastric aspirate culture for NTM-PD has been reported by 3 centers in Japan, and the positive predictive value of the gastric aspirate culture test for species-level concordance is considerably high, at approximately 85%–97% [7–9].

To enable confident use of gastric aspirate culture as an adjunct to sputum examination in NTM-PD diagnosis, further empirical research is required to confirm that NTM isolated from gastric aspirates originate from swallowed, lung-derived bacteria, rather than transient environmental bacteria present in the gastrointestinal tract. Because NTM exhibit substantial strain-level diversity, species-level concordance is insufficient and strain-level identity is required. To test this hypothesis, we conducted a retrospective observational study using core genome multi-locus sequence typing (cgMLST) [10] for species and subspecies identification and digital variable number tandem repeat (dVNTR) [11] for strain typing, to evaluate whether mycobacterial strains recovered from gastric aspirate and sputum cultures were identical. Demonstrating strain-level concordance would support the use of gastric aspirate as an alternative diagnostic specimen for NTM-PD.

## METHODS

We retrospectively identified patients who underwent gastric aspirate culture for mycobacteria at NHO Osaka Toneyama Medical Center between January 2022 and January 2025. We compared mycobacterial isolates from gastric aspirate and sputum. Only patients with a positive sputum culture obtained within 1 month of the gastric aspirate collection were included; both induced and spontaneous sputum samples were included. If multiple sputum samples were collected, only the first positive isolate was analyzed. Ethical approval was obtained from the ethics committee of NHO Osaka Toneyama Medical Center (TNH-2019063).

### Gastric Aspirate Collection

Gastric aspirates were collected by nurses in the early morning after an overnight fast by nasogastric aspiration and were immediately submitted to the laboratory for mycobacterial testing.

### Mycobacterial Culture

Sputum and gastric aspirate samples were decontaminated and then inoculated into a Mycobacterial Growth Indicator Tube (Becton Dickinson, Franklin Lakes, NJ, United States) or Ogawa medium (Kyokuto Pharmaceutical Industrial Co., Ltd., Tokyo, Japan). The cultures were incubated at 37°C until growth was detected, and the resulting isolates were subsequently used for cgMLST and dVNTR.

### Core Genome Multi-locus Sequence Typing

Subspecies-level comparison was conducted using cgMLST, as described previously [9]. DNA was extracted from mycobacterial isolates cultured from both gastric aspirate and sputum using the DNeasy PowerSoil Pro Kit (QIAGEN, Baarn, the Netherlands) and sequenced using P2 Solo instrument with the Native Barcoding Kit 96 V14 and R10.4.1 flow cells (Oxford Nanopore Technologies, Oxford, United Kingdom). Sequence data were analyzed using the mlstverse software (version 0.2.1) with an MLST database for *Mycobacterium* based on 184 genes.

### Digital Variable Number Tandem Repeat

When the isolates were identified as the same subspecies, strain-level comparison was conducted using the dVNTR method, as described previously [11]. Reported primer sets for 15 loci in *M. avium*, 16 in *M. intracellulare*, 9 in *M. abscessus*, 17 in *M. kansasii*, and 24 in *M. tuberculosis* were used [12–16]. Genome sequences were assembled with Flye (v2.9.5), and variable number tandem repeat (VNTR) loci were identified by aligning conventional VNTR primer sets using EMBOSS primersearch (v6.6.0). Product sizes were calculated, and repeat numbers were determined by rounding to the nearest integer. VNTR types were defined as a difference of one or more repeats at any locus. The results were also confirmed using conventional polymerase chain reaction-based VNTR analysis.

## RESULTS

During the study period, 277 patients were identified. Among them, 89 had a positive gastric aspirate culture, and 54 of the 89 also had a positive sputum culture within 1 month. Two patients in whom *Mycobacterium paragordonae*, which lacks established VNTR genotyping schemes, was detected in both gastric aspirate and sputum cultures were excluded; finally, 52 patients were included in the analysis (Supplementary Figure 1).

In 11 patients, *M. tuberculosis* was detected in both the gastric aspirate and sputum samples. Digital VNTR analysis showed strain-level concordance between the isolates cultured from both gastric aspirate and sputum in all patients (Supplementary Figure 2), indicating that mycobacteria detected in gastric aspirate cultures originate from the airways, which justifies using gastric aspirate culture in routine tuberculosis practice.

Nontuberculous mycobacteria were detected in both gastric aspirate and sputum samples in 41 patients. Among these, 32 patients were tested for suspected NTM-PD and 9 for suspected tuberculosis, regardless of their ability to produce sputum. The cgMLST analysis revealed subspecies-level discordance in only 1 patient, where gastric aspirate culture detected *M. avium* and sputum culture identified *M. intracellulare*. Among 40 patients in whom cgMLST analysis revealed subspecies-level concordance, dVNTR analysis showed 5 cases of strain-level discordance (Supplementary Figure 2). Table 1 shows the comparison of the baseline characteristics between the group where the NTM strains isolated from gastric aspirate and sputum cultures were identical (concordant, n = 35) and the group in which they were not (discordant, n = 6). Twenty-nine (83%) and 4 (67%) patients in the concordant and discordant groups, respectively, met the diagnostic criteria of the American Thoracic Society (ATS), European Respiratory Society (ERS), European Society of Clinical Microbiology and Infectious Diseases (ESCMID), and Infectious Diseases Society of America (IDSA) guidelines [1] within 1 year of gastric aspirate collection and were definitively diagnosed with NTM-PD. When we included cases diagnosed clinically by attending physicians, 34 (97%) in the concordant group and 6 (100%) in the discordant group were confirmed to have NTM-PD. For cases meeting the international diagnostic criteria, the median number of sputum examinations performed and time required for diagnosis were 2 (interquartile range [IQR], 2–3) and 15 (IQR, 10–23) days in the concordant group and 4 (IQR, 3–4) and 33.5 (IQR, 10.5–173.5) days in the discordant group, respectively.

**Table 1.** Characteristics of Patients With NTM

Characteristic	Overall (n = 41)	Concordant (n = 35)	Discordant (n = 6)
Age, years	72 [60, 80]	71 [58, 79]	78 [72, 87]
Sex, female	24 (59%)	21 (60%)	3 (50%)
BMI	20.3 [18.1, 22.6]	20.1 [18.2, 22.7]	20.8 [17.1, 22.1]
CT pattern			
NB type	34 (83%)	28 (80%)	6 (100%)
FC type	7 (17%)	7 (20%)	0 (0%)
Cavitary lesions	12 (29%)	11 (31%)	1 (17%)
NTM species (sputum)			
*M. avium*	23 (56%)	18 (51%)	5 (83%)
*M. intracellulare*	15 (37%)	14 (40%)	1 (17%)
*M. abscessus*	2 (5%)	2 (6%)	0 (0%)
*M. kansasii*	1 (2%)	1 (3%)	0 (0%)
NTM-PD diagnosis			
Guideline-based	33 (80%)	29 (83%)	4 (67%)
Clinically diagnosed	7 (17%)	5 (14%)	2 (33%)
Suspected	1 (2%)	1 (3%)	0 (0%)
Gastric smear positive	22 (54%)	18 (51%)	4 (67%)
Sputum smear positive	21 (51%)	18 (51%)	3 (50%)
Anti-GPL-core IgA positive (n = 31)	25/31 (81%)	24/29 (83%)	1/2 (50%)

Data are presented as n (%) or median (interquartile range).

Abbreviations: BMI, body mass index; CT, computed tomography; FC, fibrocavitary; GPL, glycopeptidolipid; NB, nodular bronchiectatic; NTM, nontuberculous mycobacteria; NTM-PD, nontuberculous mycobacterial pulmonary disease.

## DISCUSSION

Our study demonstrated that bacterial strains isolated from gastric aspirate and sputum cultures were identical in 100% of patients with *M. tuberculosis* (n = 11) and in 85% of those with NTM (n = 41) (Supplementary Figure 3). This high concordance rate suggests that, as in the case with *M. tuberculosis*, the NTM strains detected in gastric aspirate also originate from pulmonary infectious lesions, at least in most cases, rather than representing transient contamination from the environment. This finding suggests that gastric aspirate culture can be used as an adjunct in cases where sputum cultures are less likely to yield positive results for NTM-PD diagnosis.

According to the diagnostic criteria for NTM-PD in the ATS/ERS/ESCMID/IDSA guideline, positive culture results of the same NTM species (or subspecies in the case of *M. abscessus*) from at least 2 separate expectorated sputum samples are recommended to exclude the possibility of environmental contamination [1]. However, to meet the original purpose, strain-level concordance is required because *M. avium*, *M. intracellulare*, and *M. abscessus* are ubiquitous environmental organisms [1]. The strain-level identity in our study suggests that gastric aspirate culture can be reliable and that its positive results are often consistent with those from sputum for NTM-PD diagnosis.

Our genomic data provide critical molecular validation for the use of gastric aspirate. The demonstration of strain-level concordance indicates that combining 1 positive sputum with 1 positive gastric aspirate is microbiologically comparable to obtaining 2 positive sputum cultures. With a reported sensitivity of 64%–82%, this method serves as a practical alternative for patients unable to expectorate or those with paucibacillary disease [7, 8]. This approach reduces the need for repeated sputum collection and invasive bronchoscopy, enabling earlier diagnosis and timely treatment [9]. Provisional diagnostic criteria including gastric aspirate have already been proposed in Japan, specifically using the combination of 1 positive sputum and 1 positive gastric aspirate culture [17]. Collectively, these findings suggest that future international diagnostic criteria of NTM-PD could be revised to formally recognize gastric aspirate as a standard specimen.

The current Japanese provisional criteria also include the combination of a single sputum culture and serum anti-GPL-core IgA antibody positivity [17]. Moreover, a recent study further suggests that combining a gastric aspirate culture with this antibody is likewise effective [9]. Future research should evaluate the diagnostic performance of this gastric-antibody approach to facilitate earlier diagnosis when sputum samples are unavailable.

This study has several limitations. First, this was a single-center retrospective cohort study, which limits the generalizability of the findings. Second, our dVNTR analysis was based on bulk analysis of cultured isolates and targeted only the dominant strain, potentially overlooking coexisting minor strains. Considering that NTM-PD often involves polyclonal and mixed infections, particularly the nodular bronchiectatic type [18, 19], whether the observed 15% discordance reflects minor pulmonary populations missed in specimens or represents environmental contamination remains unclear. Third, the feasibility of gastric aspirate collection depends on institutional resources and clinical practices, which may vary across different facilities or regions. Future international multicenter prospective cohort studies using metagenomic analysis, which can capture polyclonal or mixed infections, are warranted to account for geographical variations and distinguish intrapatient strain diversity from environmental contamination.

In conclusion, we demonstrated that NTM strains isolated from gastric aspirate are genetically identical to those from sputum, validating their pulmonary origin. This molecular evidence supports the use of gastric aspirate as a microbiologically reliable specimen for the diagnosis of NTM-PD.

## Supplementary Material

ofag175_Supplementary_Data

## Supplementary Data

Supplementary materials are available at *Open Forum Infectious Diseases* online. Consisting of data provided by the authors to benefit the reader, the posted materials are not copyedited and are the sole responsibility of the authors, so questions or comments should be addressed to the corresponding author.
