# Supplementary material for: Gastric Aspirate Isolate Demonstrates Strain-Level Concordance With Sputum Isolate in Nontuberculous Mycobacterial Pulmonary Disease
Source: Open Forum Infect Dis. 2026 Mar 27;13(4):ofag175. doi: 10.1093/ofid/ofag175 (PMC13059685; doi:10.1093/ofid/ofag175)
Supplement: ofag175_Supplementary_Data [file ofag175_supplementary_data.docx]

**SUPPLEMENTARY FIGURES**

**Figure S1**

Figure S1: Flow diagram of total analytic sample selection

Abbreviations: NTM, nontuberculous mycobacteria; TB, tuberculosis; VNTR, variable number tandem repeat

ALT TEXT: Flowchart of patient selection showing 277 enrolled, 89 gastric aspirate positive, 54 sputum positive within 1 month, exclusion of two with *M. paragordonae*, and a final cohort of 52 patients.

**Figure S2**
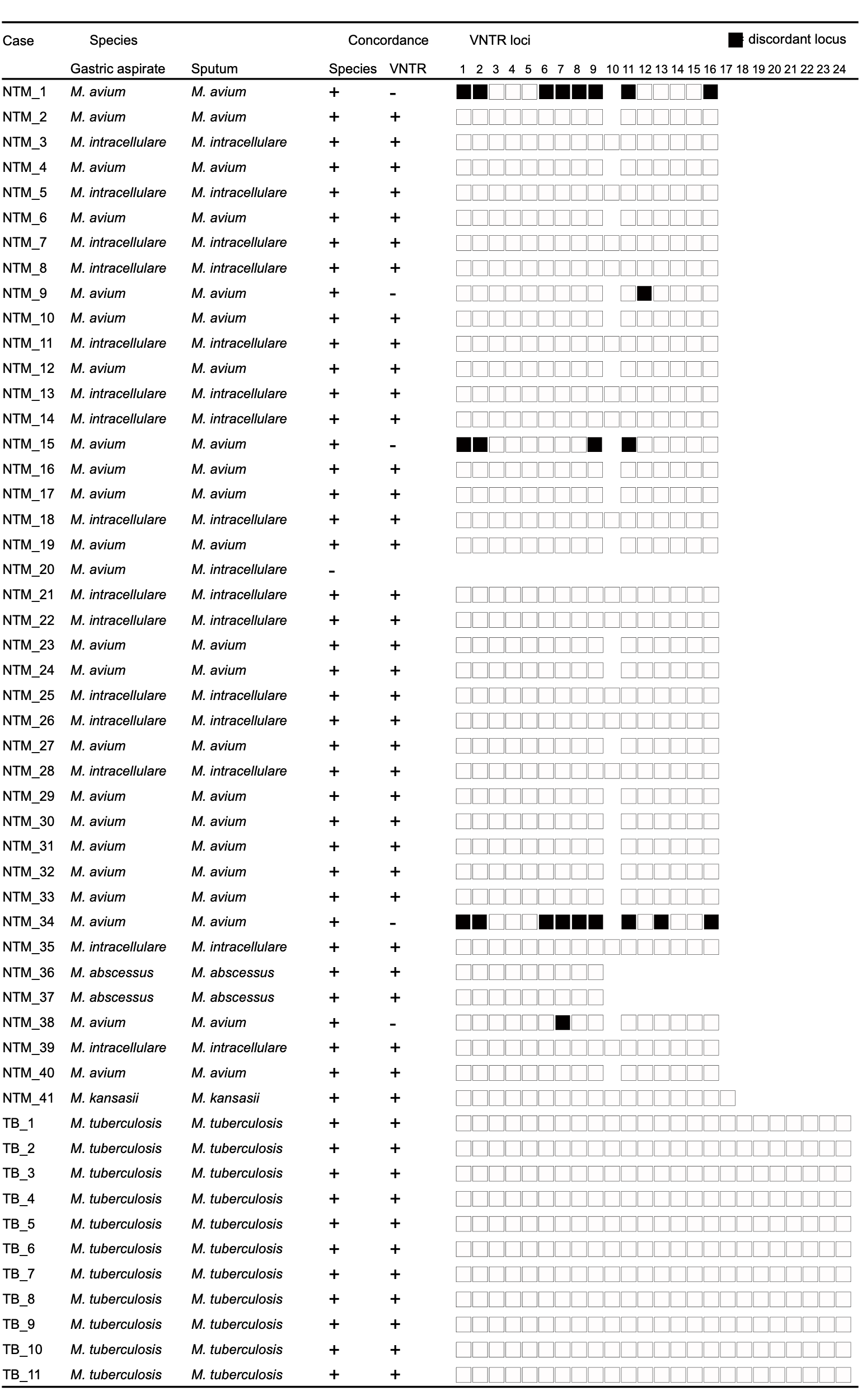


Figure S2: Mycobacterial species isolated from gastric aspirate and sputum samples, and their concordance in species identification and VNTR strain typing

Each row represents one patient. Concordance between gastric aspirate and sputum samples was assessed at both the species and VNTR levels, denoted by “+” (concordant) or “−” (discordant). For VNTR analysis, each locus from the gastric aspirate isolate is denoted by a square; the white squares indicate loci that matched those in the sputum isolate, and the black squares indicate mismatches. The number of VNTR loci analyzed was 15 for *Mycobacterium avium*, 16 for *M. intracellulare*, 9 for *M. abscessus*, 17 for *M. kansasii*, and 24 for *M. tuberculosis*.

Abbreviations: NTM, non-tuberculous mycobacteria; TB, tuberculosis; VNTR, variable number of tandem repeats

ALT TEXT: Patient-level matrix shows species and VNTR profiles with concordance. TB shows strain concordance across samples. In NTM, one species mismatch and five strain mismatches are seen, leaving 35 strain-concordant cases.

**Figure S3**


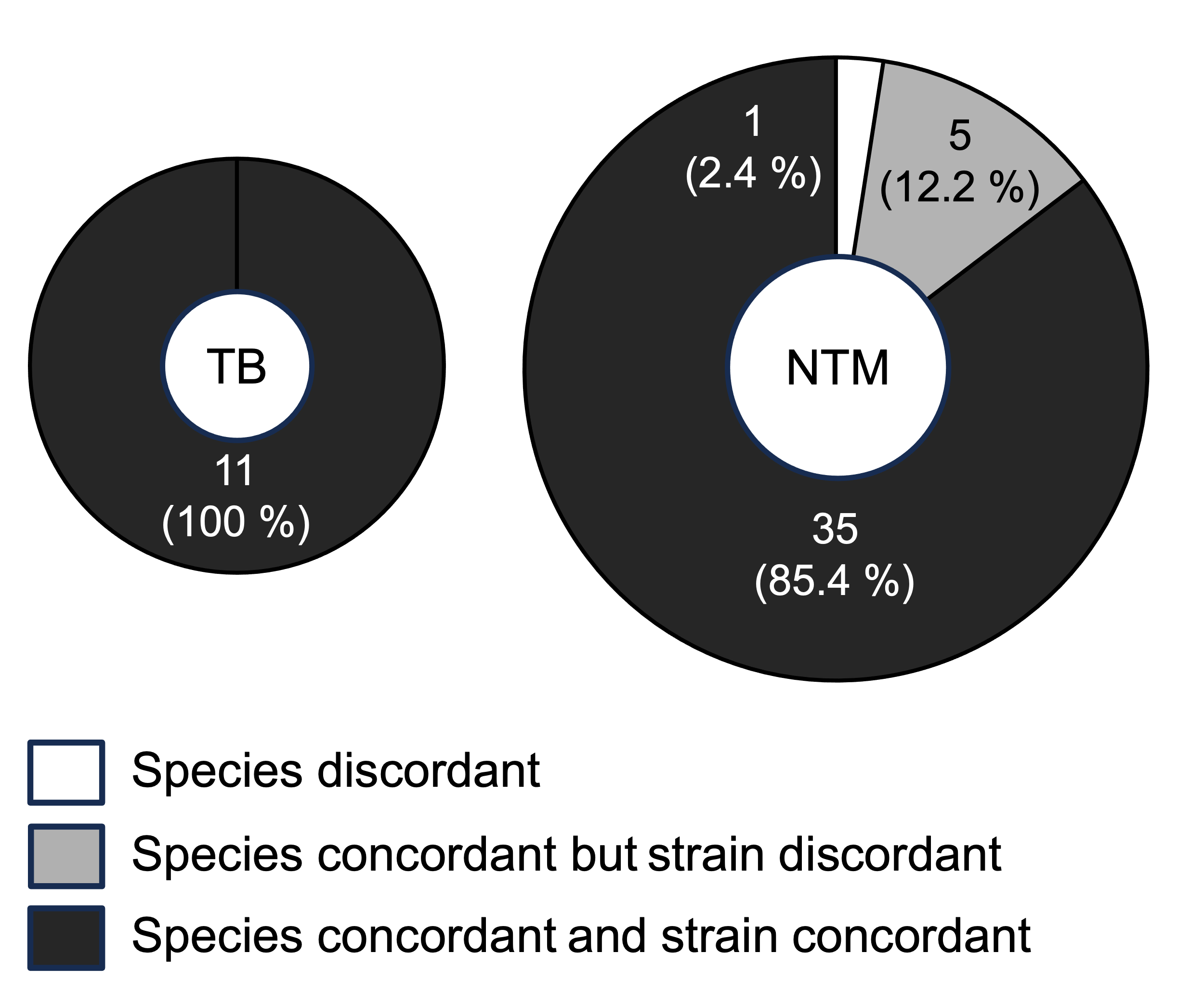


Figure S3: Species and strain-level concordance between gastric aspirate and sputum isolates in patients with tuberculosis (TB) and non-tuberculous mycobacteria (NTM)

ALT TEXT: Pie charts of species and strain concordance between gastric aspirate and sputum isolates. Strain concordance is 100% in tuberculosis and 85% in NTM pulmonary disease.
